# Self-Ligating Versus Conventional Brackets: A Narrative Review

**DOI:** 10.7759/cureus.81499

**Published:** 2025-03-31

**Authors:** Manvi A Arora, Alap Shah, Dhaval Somani

**Affiliations:** 1 Department of Orthodontics and Dentofacial Orthopedics, Karnavati School of Dentistry, Ahmedabad, IND; 2 Department of Orthodontics and Dentofacial Orthopedics, College of Dental Sciences and Research Centre, Ahmedabad, IND

**Keywords:** conventional brackets, cost, efficiency, friction, self-ligating brackets, torque expression

## Abstract

Orthodontic treatment has seen remarkable progress, particularly in bracket technology, enhancing efficiency, comfort, and oral hygiene. Self-ligating brackets (SLBs) have emerged as an alternative to conventional brackets (CBs), with claims of lower friction, shorter treatment time, better periodontal health, and reduced chairside visits. However, clinical studies yield mixed results, sparking ongoing debate. This narrative review examines the differences between SLBs and CBs in biomechanics, treatment duration, patient comfort, periodontal health, clinical efficiency, cost-effectiveness, and long-term stability based on recent research. The findings suggest that while SLBs offer certain benefits, they do not significantly surpass CBs in key treatment outcomes.

## Introduction and background

Fixed orthodontic appliances have been the foundation of orthodontic treatment for over a century. The term "bracket" came into use when Dr. Edward Angle introduced the Ribbon Arch appliance in 1916. The brackets, in particular, play a crucial role in transmitting forces from the archwire to the teeth to achieve the desired movements. The individual brackets are bonded on the tooth surface, and the wire is ligated; through this bracket and wire interface, force transmission occurs, leading to the movement of the teeth. The two primary types of brackets used today are conventional brackets (CBs) and self-ligating brackets (SLBs).

**Figure 1:**
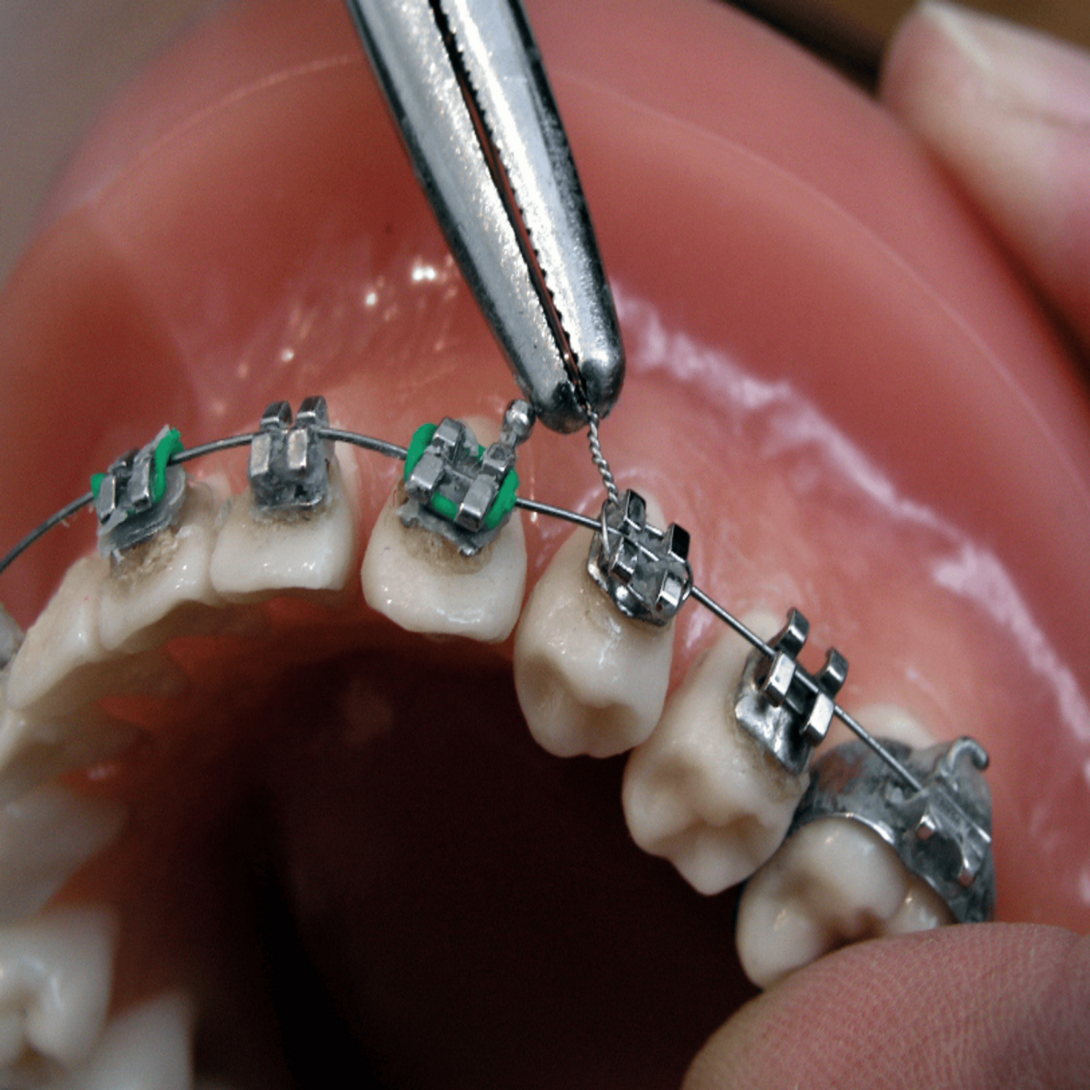
Conventional bracket Image Credit: WikiMedia Commons [1]; licensed under the Creative Commons CC BY-SA 3.0 Attribution-Share Alike 3.0 Unported license (https://creativecommons.org/licenses/by-sa/3.0/deed.en)

A CB is considered a type of bracket that requires elastomeric or metal ligatures to secure the archwire within the bracket slot (Figure 1).

**Figure 2:**
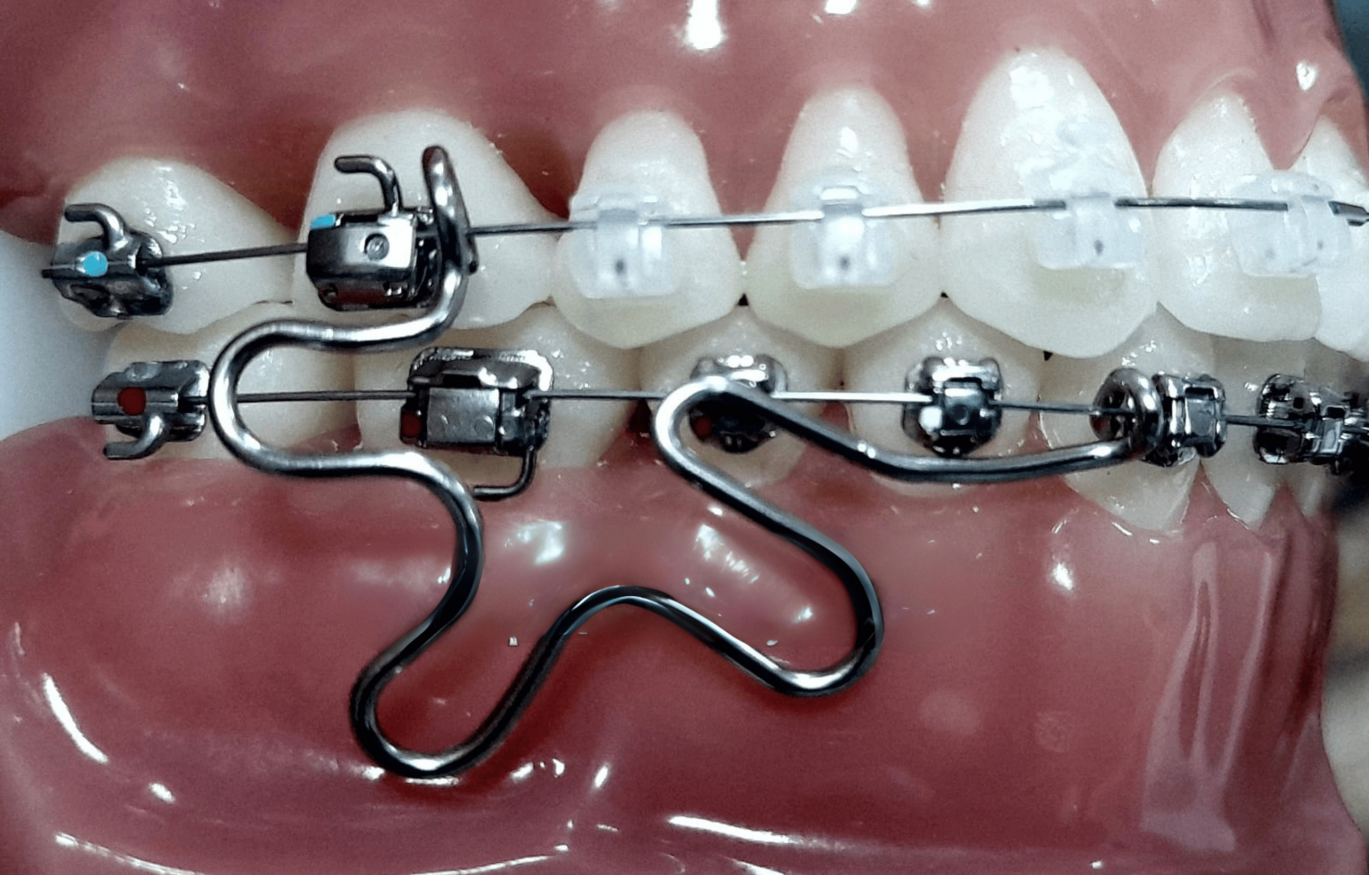
Self-ligating bracket Image Credit: WikiMedia Commons [4]; licensed under the Creative Commons Attribution-Share Alike 4.0 International license (https://creativecommons.org/licenses/by-sa/4.0/deed.en)

An orthodontic bracket that has a built-in, adjustable mechanism to secure the archwire without the need for extra ligatures is called an SLB. With a mechanical element that seals off the edgewise slot, it operates as a ligature-free system. A clipping mechanism in these brackets secures the archwire in place while granting it greater mobility, potentially resulting in a smoother tooth (Figure 2) [2,3]. 

As an alternative to the CB system, a variety of SLB systems have gained popularity in the field of orthodontics. The manufacturers and proponents of SLB systems claim these brackets provide several benefits that set them apart from traditional brackets. One of the primary benefits associated with SLB is the reduction of friction between the archwire and the bracket, which allows efficient tooth movement [5-7]. Moreover, complete engagement of the archwire by the SLB system is believed to contribute to the efficient alignment of teeth and quicker closure of spaces between them. The SLB system is divided into passive, active, and interactive brackets. This classification is based on the interaction of the wire with the bracket slot. Self-ligating active brackets contain a spring clip that stores energy and exerts pressure on the archwire, helping to control its rotation and torque.

In traditional orthodontic treatment plans, tooth extractions are often the choice to create sufficient space for the proper alignment of the teeth. However, SLB manufacturers suggest that their systems help optimize space utilization without requiring such invasive procedures. As per Damon's philosophy, a claim made by researchers is that the mechanics of self-ligation promote greater expansion of the dental arch while minimizing the forward inclination of the incisors. As a result, this approach is thought to reduce the need for tooth extractions when addressing dental crowding [8].

Apart from these structural and functional advantages, SLB systems are also promoted for their ability to enhance the overall treatment experience for both patients and orthodontists. There is a reduced need for manual adjustments, which translates to fewer interventions by orthodontic professionals. This efficiency can lead to shorter and less frequent adjustment appointments, ultimately reducing the overall duration of treatment. These brackets also contribute to improved oral hygiene. Unlike CBs, SLBs eliminate the need for these ties, which can accumulate plaque and bacteria. This design makes it easier for patients to maintain good oral hygiene, reducing the risk of cavities and periodontal problems during orthodontic treatment. Since SLB treatment is believed to involve less discomfort and a shorter treatment duration, patients may be more likely to adhere to their orthodontic care plan. Greater acceptance of treatment could lead to better outcomes, as patient cooperation plays a crucial role in the success of orthodontic procedures [6,9].

Overall, the orthodontic market has seen a flooding number of SLB options, with manufacturers continuously refining and redesigning the brackets to enhance efficiency and patient experience. While the claims surrounding SLB suggest multiple benefits over CBs, ongoing research and clinical evaluations continue to assess the extent of these advantages to determine the true impact of SLB on orthodontic treatment outcomes.

**Figure 3:**
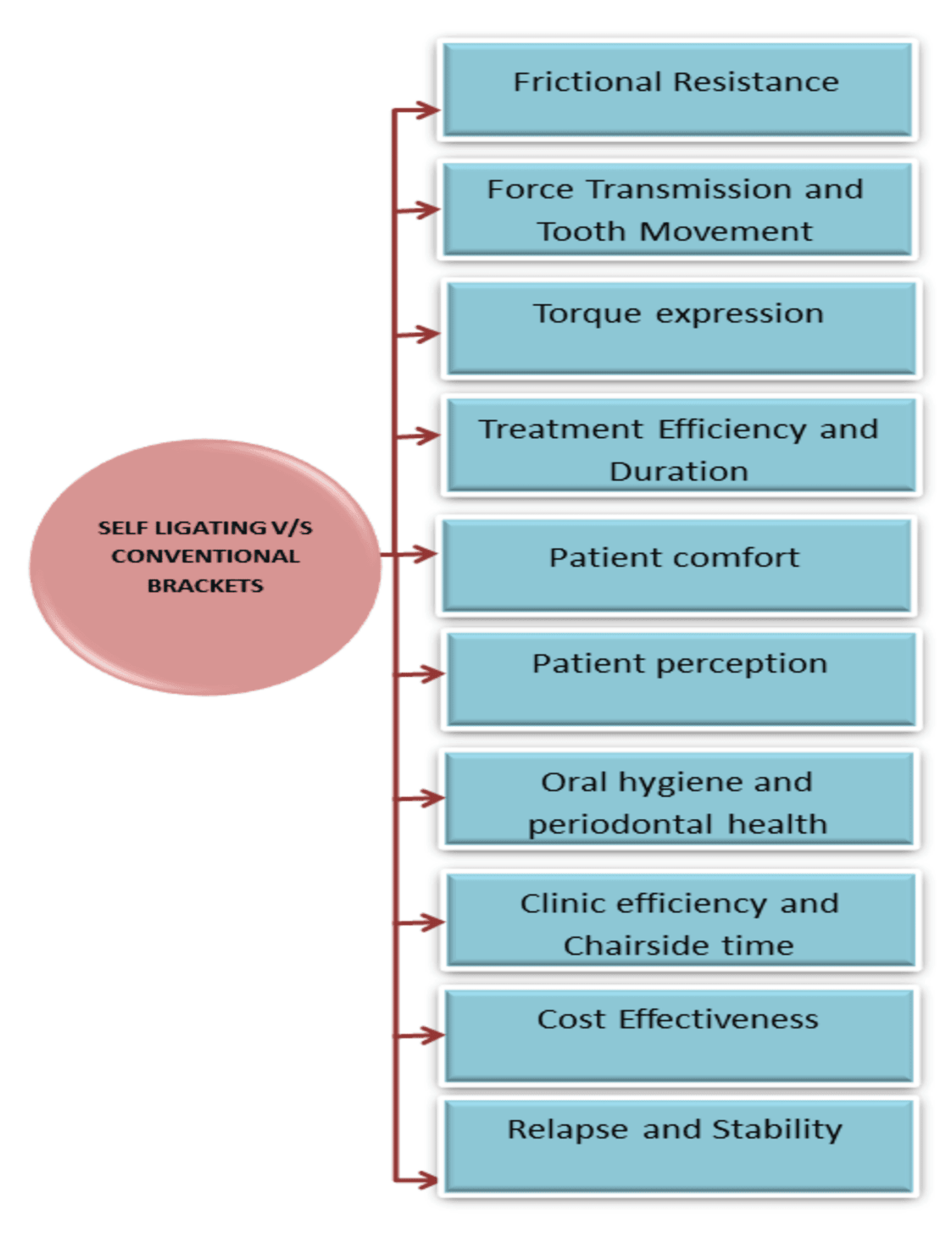
Flowchart showing the factors compared between self-ligating and conventional brackets Image Credit: Dr. Manvi A. Arora

This review critically examines the evidence comparing SLBs and CBs in terms of efficiency, biomechanics, patient experience, and long-term stability (Figure 3).

## Review

Biomechanical differences between SLBs and CBs

Frictional Resistance

The extensive use of sliding mechanics in orthodontic treatment has sparked significant research into the frictional forces that develop between the archwire and the bracket [10]. This friction is responsible for bringing resistance to tooth movement, making the alignment process difficult and less efficient to achieve [11]. As a result of friction, greater retraction forces are required to achieve the tooth movement, which can place additional strain on the anchor units [12]. This additional strain can lead to undesirable side effects, such as unwanted tooth movement in areas meant to remain stable [12,13]. Considering these frictional forces is crucial for optimizing orthodontic treatment outcomes, reducing treatment time, and minimizing adverse effects [14].

**Figure 4:**
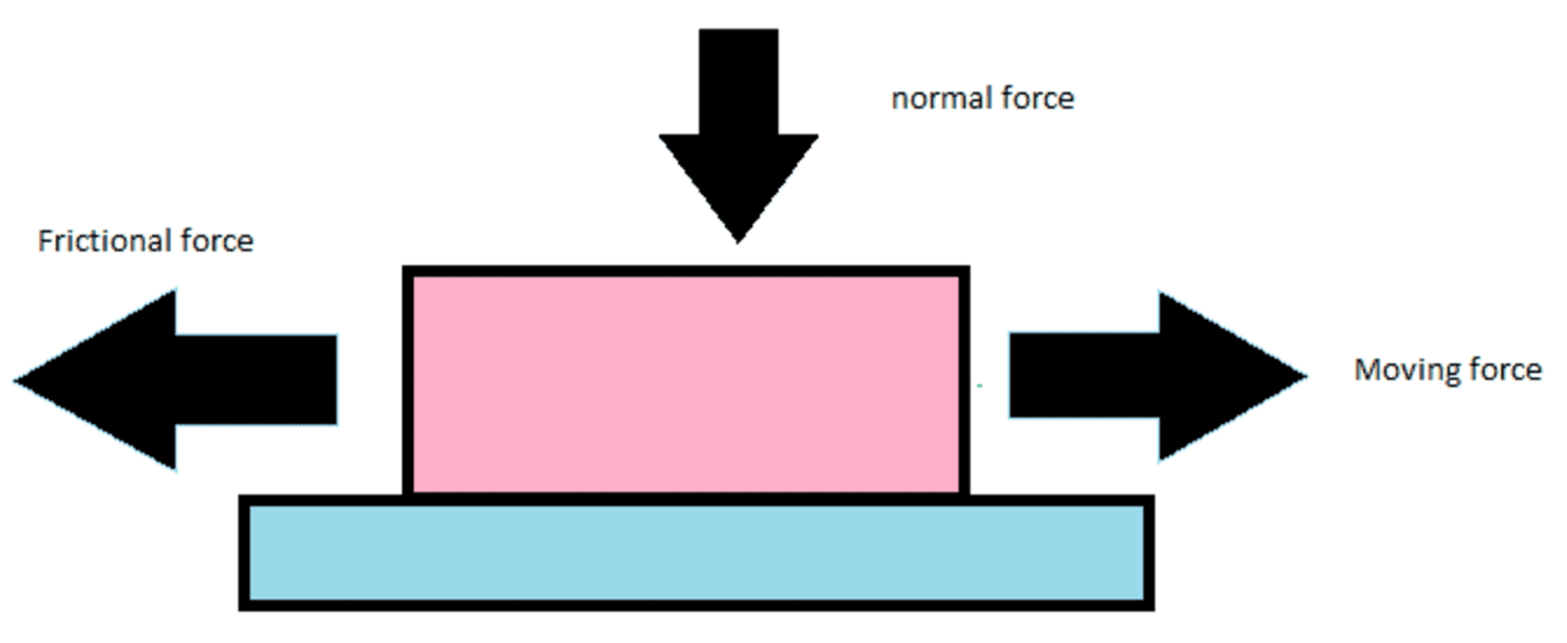
Frictional force acting on the block showing an example of moving force, normal and frictional Image Credit: Dr. Manvi A. Arora

Friction is defined as the force that opposes movement between two surfaces in contact when an external force is applied, causing one surface to glide over the other (Figure 4) [15,16]. A number of factors affect friction, including the dynamics of movement between the surfaces, the strength and direction of applied forces, and external influences like lubrication and temperature [17]. Surface roughness and material properties also play important roles in determining the level of friction [17].

**Figure 5:**
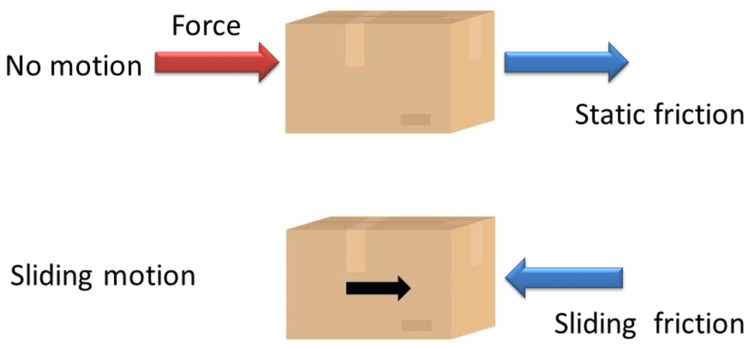
Two types of friction: static and kinetic (sliding) friction Image Credit: Dr. Manvi A. Arora

Friction can be understood under two types: static friction and kinetic friction. Static friction refers to the force that is required to initiate the movement of the body, whereas kinetic friction (sliding friction) refers to the force that is required to resist the continuous motion of the body (Figure 5). 

**Figure 6:**
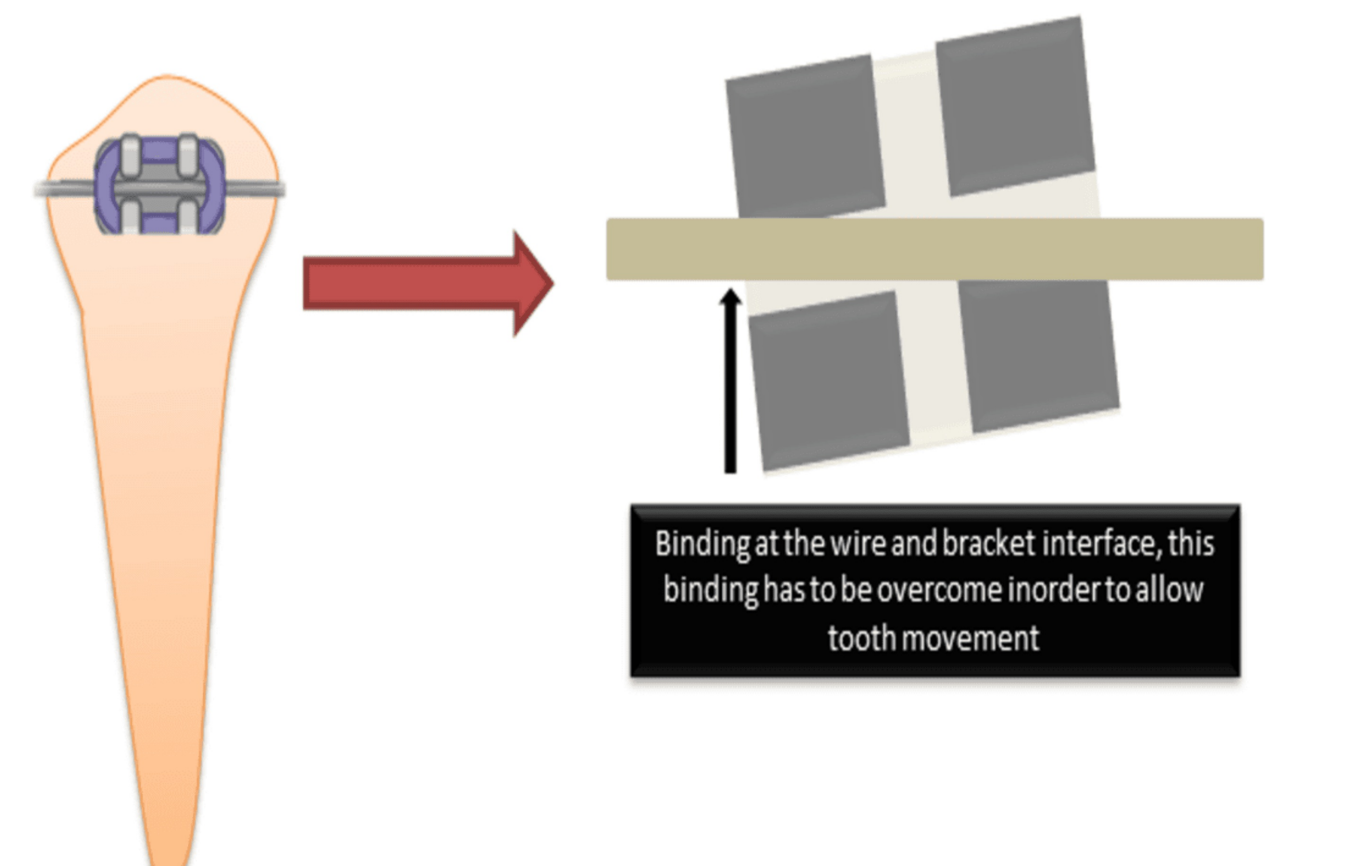
Friction and binding in a bracket Image Credit: Dr. Manvi A. Arora

According to various research, the coefficient of static friction is always higher than that of kinetic friction [16,18]. In orthodontics, static friction is considered more significant in influencing tooth movement. When a tooth moves along an archwire, it does not slide smoothly but rather progresses in small, incremental jumps (Figure 6). Each movement must overcome static friction and biological resistance in order to upright the root within the alveolar bone. This process highlights the importance of understanding and managing frictional forces in orthodontic treatments to enhance efficiency and reduce unnecessary resistance to tooth movement [18].

A comprehensive comparative analysis between SLBs and CBs, focusing on the frictional resistances during orthodontic treatment, demonstrated that passive SLBs exhibited significantly lower frictional resistance compared to CBs [19]. This reduction in friction is primarily due to the absence of elastic or metal ligatures. In CBs, ligatures are used to secure the archwire tightly within the bracket slot, thereby increasing frictional forces that can hinder tooth movement. SLBs have a built-in clip or gate mechanism to hold the archwire in place, thus eliminating the need for additional ligatures [20]. This design reduces binding and notching effects and also facilitates a more controlled and efficient sliding mechanism along the archwire. As a result, tooth movement occurs with less resistance, which can lead to a more predictable and potentially faster alignment phase in orthodontic treatment. The reduced frictional forces in SLBs contribute to decreased treatment time, reduced need for heavy force applications, and improved patient comfort [21]. The increased biomechanical efficiency of SLBs makes them advantageous in cases requiring significant tooth movement, such as leveling and aligning the crowded dentition. However, the researchers also emphasized that while SLBs offer prospective benefits in terms of friction reduction, their clinical performance eventually depends on numerous parameters, including wire size and substance, bracket slot diameters, and the specific mechanics utilized during therapy [22].

Kusy and Whitley investigated the dynamics of frictional forces within various bracket systems, focusing on SLBs and CBs [23]. Their research attempted to quantify and compare the resistance experienced by archwires as they slide through both bracket systems, a significant component impacting the effectiveness of tooth movement during orthodontic treatment. SLBs, particularly passive SLBs, permit significantly smoother wire movement compared to CBs [23]. The study emphasized on the fact that reduced friction associated with SLBs can contribute to more effective alignment and leveling phases during orthodontic treatment [23,24]. By minimizing unnecessary resistance, SLBs enable lighter forces to be applied while still achieving significant tooth movement. This could theoretically result in a shorter treatment duration and greater patient comfort compared to CBs, which often require higher force applications to overcome frictional resistance. However, despite these potential advantages, Kusy and Whitley also acknowledged that the actual clinical performance of SLBs is influenced by a variety of external and intraoral factors which includes the specific design and material composition of the brackets, the type and dimension of the archwires used, the degree of torque and angulation present in the system, and individual patient-related variables such as oral hygiene, occlusal forces, and saliva composition [23].

Frictional forces can also be influenced by different bracket-archwire configurations [25]. Monteiro et al. evaluated two types of brackets, SLBs (SmartClip, 3M/Unitek, Maplewood, Minnesota, United States) and CBs (Gemini, 3M/Unitek, Maplewood, Minnesota, United States), with three types of archwires: nickel-titanium (NiTi), beta-titanium (β-Ti), and stainless steel (SS) [26]. Brackets were tested at different slot angulations of 0°, 5°, and 10°, and frictional forces were measured as the wires were pulled through the bracket slots. The results showed that SLBs generally produced lower frictional resistance than CBs across all tested conditions. However, there was an increase in frictional resistance as the bracket-slot angulation increased for both bracket types. The SS wires exhibited the least frictional forces in CBs, followed by NiTi and β-Ti wires.

As mentioned above, the SLBs are available in active and passive bracket systems, which also show differences in frictional resistance. A study by Krishnan et al. showed that SLBs do show lower frictional values than CBs, but there was no difference in frictional value between active and passive SLBs when SS rectangular wire was used. When NiTi and β-Ti rectangular wires were used, the active self-ligating system showed more frictional values [27]. 

Force Transmission and Tooth Movement

**Figure 7:**
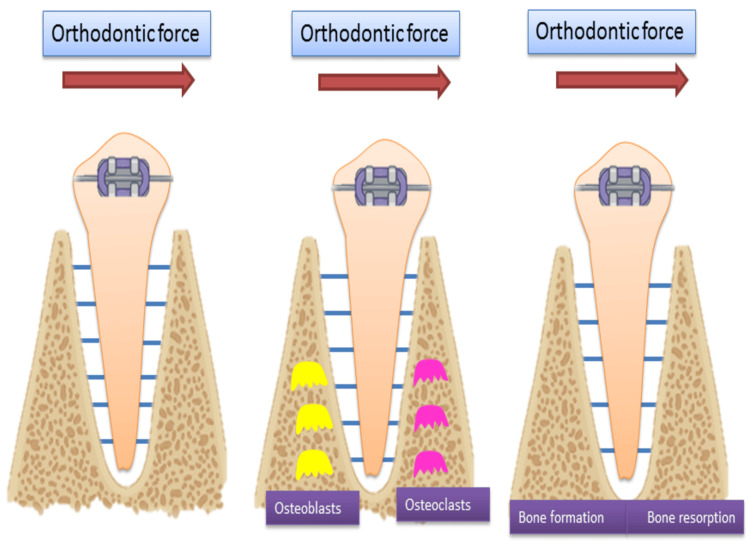
Response to orthodontic tooth movement Image Credit: Dr. Manvi A. Arora

Orthodontic tooth movement is a biological process that happens when brackets or other orthodontic devices apply forces to the teeth [28]. These forces play a crucial role in shifting the teeth into better positions, enhancing both their function and appearance. It occurs through the remodelling of the alveolar bone in response to applied mechanical forces. When orthodontic forces are applied to a tooth, the load is transferred from the tooth through the periodontal ligament (PDL) to the surrounding alveolar bone [29]. This process causes slight, reversible damage to the periodontium that holds the tooth in place. This minor injury, combined with the physical forces on the PDL, triggers selective bone remodelling at different areas around the tooth. These areas are called "tension" and "compression," based on the forces involved and the tissue response observed [30]. On the tension side, the movement of the tooth root creates strain on the PDL fibres that connect the tooth to the bone. Conversely, on the compression side, the PDL experiences a compressive force as the tooth moves toward the bone, causing the PDL fibres to unload [31]. This mechanical stress is translated into a biological response, where bone resorption occurs on the compression side and bone formation takes place on the tension side. Interestingly, this is different from the response seen in other bone areas, where pressure typically stimulates bone formation (Figure 7) [31-35]. 

Optimal orthodontic force is the amount of force that stimulates efficient tooth movement while minimizing tissue damage, root resorption, and patient discomfort [33,36,37]. This force should induce biological responses in the PDL without causing excessive pressure or necrosis. The ideal force should be continuous and of light magnitude. Increasing the force level would likely heighten the risk of side effects, including external apical root resorption (EARR), uncontrolled tipping, and increased hyalinization, all while compromising patient comfort and clinical efficiency [38-40].

Some researchers suggest that lower friction in SLBs results in improved force transmission and more physiologic tooth movement. However, the clinical relevance remains uncertain. Pandis et al. investigated the differences in force exerted by SLBs and CBs during orthodontic alignment [41]. Using a laboratory simulation, the study measured the forces produced when engaging an archwire with different bracket systems. The results showed that the force magnitude varies depending on factors such as interbracket distance, ligation method, and the number of teeth engaged in the proximal and distal regions of the arch [41-45]. Additionally, the study highlights that the relaxation of ligatures in CBs and the unique clip mechanism in SLBs influence the amount of force transmitted to teeth over time [46]. However, while SLBs are often associated with lower friction, the study suggests that this does not necessarily translate to clinically significant differences in alignment forces [41,47]. When Damon3 (Ormco, Glendora, California, United States) and CBs were compared by Scott et al. regarding the force levels in initial alignment, it was concluded that Damon3 delivered low forces only in the initial alignment phases which was not significant [48].

Torque Expression

The twisting of a structure around its longitudinal axis, producing an angle of twist, is called torque [49]. It is a shear-based movement that causes rotation. It is an orthodontic adaptation that describes rotation perpendicular to the tooth's long axis and denotes the labiolingual crown/root inclination of a tooth [50,51]. The ideal labiolingual inclination of the anterior and posterior teeth is thought to be crucial in clinical orthodontics in order to provide a good occlusal connection, a beautiful smile line, appropriate root motions, and consequently long-lasting stability of the orthodontic result [52]. There is a notable difference between what has been achieved in theory and what is observed in clinical settings when it comes to the bracket/wire. There are numerous reasons for these torque variances, including bracket design, wire-to-slot clearance (or play), slot dimension, wire stiffness, torsion magnitude, bracket deformation, ligature mode, and wire size [53,54]. In comparison to SLBs, CBs show more torque expression [55,56]. Among active and passive SLBs, the latter ones show maximum torque expression [56]. This occurs because, particularly as the arch's diameter increases, the clip continuously forces the wire against the bracket slot. They further assert that the active self-ligating ones have the capacity to realign themselves in three dimensions until the arch is fully inserted into the slot and apply a constant push to the arch, improving orthodontic movement precision [57].

Treatment efficiency and duration

During an initial consultation, clinicians are often required to address questions about the estimated duration of the proposed treatment. The response typically depends on various factors, including the clinician's experience, which may be influenced by their educational background, technical expertise, and practice management approach [58]. Many patients are concerned about how long they will need to have brackets. Accurate predictions of treatment time can also assist clinicians in optimizing their practice management [59]. Various factors can influence the duration of treatment, including age, gender, bracket type, initial molar relationship, and whether the treatment plan involves extractions or not [60]. There has been no observed variation in the duration of orthodontic treatment based on the type of brackets utilized. Whether the brackets are metallic or ceramic or whether they are conventional or self-ligating, the overall treatment time remains consistent [60-63].

One of the main marketing claims for SLBs is that they reduce total treatment time. When Damon3 and Synthesis CBs (Ormco, Glendora, California, United States) were compared for their treatment duration, there was no difference found [64]. While SLBs may improve early alignment, their overall impact on treatment duration is minimal [41,65]. Machibya et al. did a similar comparison between SLBs and CBs with respect to duration time to treat the patients; for SLBs, it was 19.19 months, and CBs were 21.25 months, which was insignificant [66].

Canine retraction is probably the most common clinical situation where sliding mechanics is used to move a tooth over a relatively large distance. This phase of orthodontic treatment typically occurs after premolar extractions, where the canines are moved distally into the extraction space to create proper alignment and achieve an ideal occlusion. Since canines have long, strong roots and are subjected to significant forces during retraction, the efficiency and biomechanics of tooth movement are critical. da Costa Monini et al. did a split-mouth study to evaluate the time taken to retract the canine, which revealed no significant differences between SLBs and CBs in terms of the velocity of canine retraction [67]. The type of bracket (SLB or CB) does not significantly impact the efficiency of canine retraction or the maintenance of anchorage during orthodontic treatment [48,66,68-70].

Patient comfort and pain perception

Orthodontic patients are always concerned about the pain that the treatment may cause. Evaluating pain in an objective manner is quite challenging, as it is widely recognized that pain perception is shaped by a variety of influencing factors [71]. Orthodontic pain is believed to originate in the PDL due to various biological processes, including pressure, restricted blood flow (ischemia), inflammation, and swelling [72]. During orthodontic tooth movement, the levels of several inflammatory substances known to heighten pain sensitivity, such as histamine, prostaglandins, serotonin, bradykinin, and substance P, increase within the periodontium. These biochemical changes contribute to the discomfort experienced by patients undergoing orthodontic treatment [73,74].

SLBs are believed to reduce friction on the teeth, which could allow for lighter forces and potentially lessen pain in the pulp and PDL [5]. However, their effectiveness in reducing discomfort during treatment remains debated. A systematic review of high-quality studies concluded that there is currently insufficient evidence to indicate any difference in pain levels between self-ligating and conventional appliances [75]. A prospective randomized clinical trial by Pringle et al. [76] aimed to compare pain levels between two fixed orthodontic bracket systems, i.e., Damon3 and Tru Straight CB (Ormco Europe, Amersfoort, the Netherlands), during the initial phase of tooth movement. The results indicated that patients with the Damon3 SLBs reported lower mean maximum pain intensity and significantly lower mean pain intensity compared to those with the Tru Straight CBs. On the other hand, a study done by Scott et al. in 2008 showed that Damon3 did not provide any reduction in discomfort in comparison to CBs [77]. Tecco et al. also did a study on the pain perception between Damon SLII and CBs [78]. It showed that the use of analgesics was 16.5% for SLB and 10% for CB patients. The CB patients showed constant pain, whereas SLB patients showed pain associated with biting and chewing. When SmartClip and Victory System were compared for pain values, their differences were insignificant [79].

Oral hygiene and periodontal health

Orthodontic treatment ensures precise tooth alignment and optimizes the occlusion-jaw relationship. This not only enhances quality of life by improving eating, speech, and appearance but also contributes to overall health. As a result, the number of adults seeking orthodontic treatment has steadily increased in recent years. The relationship between orthodontic treatment and periodontitis prevalence has been a topic of debate among researchers. With the increasing number of adult orthodontic patients, the importance of periodontal health has gained more attention. Studies suggest a complex, often synergistic interaction between orthodontics and periodontitis. Orthodontic treatment can improve periodontal health by aligning teeth, balancing occlusion, and facilitating better oral hygiene, thereby reducing occlusal trauma. However, fixed orthodontic appliances may contribute to supragingival biofilm accumulation and negatively affect periodontal tissues. Additionally, orthodontic forces can induce inflammation in the periodontium, a necessary response for tooth movement. A key challenge in orthodontics is achieving successful treatment while minimizing adverse effects on the root and periodontium [80]. Orthodontic tooth movement has often contributed to improved periodontal health, while periodontal therapy frequently supports and facilitates orthodontic tooth movement [81].

For adult patients who have pre-existing periodontal conditions, orthodontic treatment can still be safely performed following proper periodontal stabilization [82]. Research has demonstrated that orthodontic interventions, when carried out under controlled conditions and with appropriate periodontal management, do not have detrimental effects on periodontal health [83]. In fact, properly aligned teeth can improve oral hygiene practices and contribute to better long-term periodontal stability. Studies have shown that with careful treatment planning and close monitoring by both orthodontists and periodontists, adults with periodontal concerns can undergo orthodontic treatment successfully without experiencing further periodontal deterioration [83].

Maintaining good oral hygiene during orthodontic treatment is essential, as poor hygiene can lead to side effects such as enamel demineralization, gingival inflammation, and halitosis, which can negatively impact the quality of life [84]. Oral hygiene most of the time gets complicated post-orthodontic appliance placement, which leads to increased gingivitis and dental caries [85-87].

SLBs, by eliminating elastic ligatures, are thought to reduce plaque retention compared to CBs. Wang et al. investigated the impact of SLB on periodontal health and inflammatory markers in patients with chronic periodontitis and concluded that SLBs help reduce inflammation, enhance periodontal conditions, and contribute to overall dental health improvements [88]. The study highlights the potential advantages of SLBs in minimizing periodontal stress and promoting better oral health outcomes in orthodontic patients with pre-existing periodontal concerns [88,89].

Enamel decalcification is a frequent concern in orthodontics, often resulting from plaque accumulation around brackets [90-92]. To study that, Pellegrini et al. did a randomized controlled trial and concluded that there was an increased plaque accumulation on the elastomeric ligatures than the SLBs [93]. According to Pandis et al., SLBs do not offer any advantage over CBs in terms of periodontal health [94]. Similarly, research by Shrestha et al. suggests that SLBs do not provide superior periodontal benefits compared to CB systems [95]. Apart from the bracket systems, proper hygiene control and regular cleaning of teeth can help patients secure their periodontal health.

Clinical efficiency and chairside time

The duration required to remove or ligate orthodontic wires is a factor of interest in clinical practice; however, it is considered a less significant advantage of SLB systems when compared to other key benefits, such as secure ligation and the potential for reduced friction and force generation during tooth movement. In an effort to improve clinical efficiency by cutting down on ligation time, the first SLB, that is, the Russell attachment, was created in the middle of the 1930s [96]. While SLB systems may facilitate faster archwire changes due to their clip-based design, their true value lies in their biomechanical efficiency and ability to provide more controlled tooth movement with potentially lighter forces [97].

Nevertheless, from a practical standpoint, the ease and speed of archwire manipulation play a crucial role in a busy orthodontic setting. Reducing chairside time per patient allows for greater clinical efficiency, improved workflow, and enhanced patient comfort. In high-volume orthodontic practices, where numerous patients are treated daily, the ability to quickly and effectively adjust or replace archwires can contribute to smoother appointment scheduling and increased overall productivity. Therefore, while the reduction in ligation time may not be the primary benefit of SLBs, its impact on clinical efficiency and ergonomics should not be overlooked [97]. The time required for ligation showed an average reduction of two seconds per bracket when closing slides with the Damon2 system (SDS Ormco, Orange, California, United States), while opening slides took one second less per bracket compared to the Orthos elastics (SDS Ormco, Orange, California, United States). Additionally, the average time needed for wire placement in a fully bonded arch was significantly shorter with the Damon2 system, taking only 46 seconds, whereas the Orthos brackets required 98 seconds for the same procedure [97].

A study conducted by Harradine found that while the time savings for ligation and re-ligation with the Damon SLB system were statistically significant, they were clinically minimal, averaging just 24 seconds per archwire removal and replacement [98,99]. This benefit is particularly evident in lingual orthodontic self-ligating cases with rotated teeth because the low bracket widths and interbracket spans necessitate executing multiple overties to properly engage the archwire and limit the rotation, which takes more time [100,101]. However, it is important to note that SLBs eliminate the need for a chairside assistant during the ligation process, as they do not require the operator to handle elastomeric or wire ligatures, streamlining the procedure [102]. While this may improve orthodontic practice efficiency, its impact on overall treatment duration remains inconclusive.

Cost-effectiveness 

The cost-effectiveness of an orthodontic bracket system depends on multiple factors, such as the initial price of the brackets, treatment efficiency, time spent in the dental chair, and long-term treatment results. Traditional metal brackets are typically more budget-friendly than SLBs or ceramic brackets, which usually come at a higher initial cost. However, SLBs are often advertised for their ability to shorten treatment duration and reduce the number of visits needed, potentially balancing out their higher price. Furthermore, brackets designed for easier wire adjustments and reduced friction can improve clinical efficiency, benefiting both orthodontists and patients. The overall success of treatment, including long-term stability and gum health, also impacts cost-effectiveness [103]. The bracket system and their financial value should be assessed based on treatment outcomes, patient comfort, and practice workflow rather than just their upfront cost. SLBs on the market today are generally more costly than most CBs. Many orthodontists have expressed concerns about whether the potential improvements in clinical efficiency associated with SLBs are sufficient to justify their higher price.

Prettyman et al. indicated that orthodontists showed a strong preference for CBs in terms of cost-effectiveness. A significant majority of practitioners (68%) believed that CBs offer better financial value compared to SLBs [104]. Furthermore, among the orthodontists who chose to discontinue the use of SLBs, the primary reason cited was the lack of substantial advantages over CBs that would justify the higher cost. Many practitioners felt that the perceived benefits of SLBs, such as reduced treatment time or improved efficiency, were not significant enough to warrant the additional expense, leading them to revert to the more cost-effective CB systems.

Harradine suggests that SLBs, especially the Damon system, may offer cost efficiency by reducing treatment duration through decreased friction [68]. This reduction in friction can lead to fewer required appointments, ultimately lowering overall treatment expenses. Although SLBs have a higher initial cost compared to CBs, the time saved throughout the treatment process can help balance out the increased upfront investment, making them a potentially cost-effective option in the long run.

Relapse and long-term stability

The stability of properly aligned teeth is inconsistent and often difficult to predict. As a result, maintaining dental alignment after orthodontic treatment remains a complex and ongoing challenge for orthodontists. Numerous follow-up studies on treated cases have demonstrated that while significant improvements in dental positioning can be successfully achieved through orthodontic intervention, there is a persistent tendency for teeth to gradually shift back toward their original positions over time. This phenomenon, known as relapse, may occur many years after the completion of treatment, emphasizing the need for long-term retention strategies to preserve the achieved alignment. Post-treatment stability is a critical consideration in orthodontics. A retrospective study by Yu et al. compared the long-term stability of treatments using SLBs to those using CBs [105]. The study included 60 adolescents, divided equally between the two treatment modalities, with an average follow-up period of approximately 7.5 years. The findings indicated no significant differences in long-term stability between the two groups, suggesting that SLBs do not offer superior relapse prevention compared to CBs.

Willeit et al. focused on the stability of transverse dental arch dimensions following treatment with passive SLBs over a six-year follow-up period [106]. The results demonstrated that the transverse expansion achieved during treatment was largely maintained, indicating favorable long-term stability in arch width.

Limitations and recommendations

Although the main points of the comparison between the two systems were covered in the evaluation, some outcomes are subjective to the operator. There are some confounding elements that cannot be eliminated, such as the fact that maintaining dental hygiene depends on the patient's cooperation. Even cost-effectiveness depends on the operator's perspective and how it is handled; many businesses sell less expensive bracket kits, which entice orthodontists to purchase them but result in poor performance. The patient-driven treatment method should always be recommended since it should be as effective as possible, regardless of whether a conventional or self-ligating system is utilized. This means that in order to get the best possible comfort, compliance, and results, treatment programs should be customized to meet the specific needs of each patient. In addition to increasing efficiency, a patient-centered approach also improves overall dental health and patient happiness. To optimize the efficiency of the selected orthodontic system, factors including treatment length, cleanliness maintenance, and lifestyle preferences should be taken into account. In the end, attaining effective and long-lasting benefits depends heavily on patient participation and adherence.

## Conclusions

SLBs and CBs have been extensively compared in orthodontics, with conflicting findings regarding their advantages and clinical effectiveness. While SLBs are often marketed as reducing treatment time, minimizing friction, improving oral hygiene, and enhancing patient comfort, the scientific evidence does not consistently support these claims. Studies suggest that while SLBs may slightly reduce chairside time by eliminating elastic or metal ligatures, this does not significantly shorten overall treatment duration. Although SLBs improve efficiency in wire changes and may reduce appointment frequency, their impact on total treatment time remains minimal. While SLBs are often marketed as promoting better oral hygiene due to the absence of elastomeric ligatures, patient compliance remains the key factor in maintaining periodontal health. In terms of cost-effectiveness, SLBs have a higher initial cost than CBs, and despite claims that fewer appointments may offset this expense, studies indicate only marginal financial benefits. Additionally, long-term stability and relapse rates are comparable between SLB and CB treatments, with no strong evidence favoring SLBs for superior retention. Ultimately, the choice between SLBs and CBs should be guided by individual patient needs, case complexity, and practitioner preference rather than marketing claims.
